# Circular RNA as a Potential Biomarker for Forensic Age Prediction

**DOI:** 10.3389/fgene.2022.825443

**Published:** 2022-02-07

**Authors:** Junyan Wang, Chunyan Wang, Yangyan Wei, Yanhao Zhao, Can Wang, Chaolong Lu, Jin Feng, Shujin Li, Bin Cong

**Affiliations:** ^1^ Hebei Key Laboratory of Forensic Medicine, Collaborative Innovation Center of Forensic Medical Molecular Identification, Research Unit of Digestive Tract Microecosystem Pharmacology and Toxicology, College of Forensic Medicine, Chinese Academy of Medical Sciences, Hebei Medical University, Shijiazhuang, China; ^2^ Physical Examination Center of Shijiazhuang First Hospital, Shijiazhuang, China

**Keywords:** forensic genetics, biomarkers, circular RNA, age prediction, machine learning

## Abstract

In forensic science, accurate estimation of the age of a victim or suspect can facilitate the investigators to narrow a search and aid in solving a crime. Aging is a complex process associated with various molecular regulations on DNA or RNA levels. Recent studies have shown that circular RNAs (circRNAs) upregulate globally during aging in multiple organisms such as mice and *C.elegans* because of their ability to resist degradation by exoribonucleases. In the current study, we attempted to investigate circRNAs’ potential capability of age prediction. Here, we identified more than 40,000 circRNAs in the blood of thirteen Chinese unrelated healthy individuals with ages of 20–62 years according to their circRNA-seq profiles. Three methods were applied to select age-related circRNA candidates including the false discovery rate, lasso regression, and support vector machine. The analysis uncovered a strong bias for circRNA upregulation during aging in human blood. A total of 28 circRNAs were chosen for further validation in 30 healthy unrelated subjects by RT-qPCR, and finally, 5 age-related circRNAs were chosen for final age prediction models using 100 samples of 19–73 years old. Several different algorithms including multivariate linear regression (MLR), regression tree, bagging regression, random forest regression (RFR), and support vector regression (SVR) were compared based on root mean square error (RMSE) and mean average error (MAE) values. Among five modeling methods, regression tree and RFR performed better than the others with MAE values of 8.767 years (S.rho = 0.6983) and 9.126 years (S.rho = 0.660), respectively. Sex effect analysis showed age prediction models significantly yielded smaller prediction MAE values for males than females (MAE = 6.133 years for males, while 10.923 years for females in the regression tree model). In the current study, we first used circRNAs as additional novel age-related biomarkers for developing forensic age estimation models. We propose that the use of circRNAs to obtain additional clues for forensic investigations and serve as aging indicators for age prediction would become a promising field of interest.

## Introduction

Age prediction of an unknown individual can facilitate case investigations and disaster victim identification. Estimating the age of known persons with an unclear age can provide important clues in legal affairs (Schmeling et al., 2016). The age of individuals can be determined by techniques that rely on morphological measures of teeth and skeletal remains (Ichikawa et al., 2010; Marquez-Ruiz et al., 2018). But this approach is restricted to samples with a nearly complete skeleton and is influenced by subjective factors (Schmeling et al., 2007).

In most cases, only fragmentary remains are left by the perpetrator after committing a crime. Forensic molecular methods have allowed researchers to obtain genetic information of a person from biological evidence retrieved from crime scenes (Park et al., 2016). In recent years, several molecular methods were proposed, such as telomere shortening (Mensa et al., 2019), mitochondrial DNA deletion (Zapico and Ubelaker, 2016; Yang et al., 2020), signal-joint T-cell receptor excision circle (sjTRECs) (Yamanoi et al., 2018), and DNA methylation (DNAm) (Meng et al., 2019; Dias et al., 2020; Freire-Aradas et al., 2020). Among these biomarkers, the DNAm pattern was considered as the most promising age-predictive biomarkers for forensic and clinical use due to its high prediction accuracy. In particular, Zbiec-Piekarska et al. (Zbiec-Piekarska et al., 2015a) conducted a research based on pyrosequencing data for two CpG sites in the *ELOVL2* gene and achieved relatively high prediction accuracy with a mean absolute deviation (MAD) from the chronological age of 5.03 years. Another model based on 5 CpG sites reported by Zbiec-Piekarska showed high prediction accuracy with a MAD of 3.9 years. Hence, several sites of the gene *ELOVL2* is believed to be the most hopeful locus for age prediction (Zbiec-Piekarska et al., 2015b). DNAm-based age prediction methods can be developed in a way with a relatively high accuracy for individual age estimation. However, it cannot be denied the DNAm method suffers from several challenges: The DNAm change pattern can be greatly influenced by several factors such as smoking, nutrition, and diverse diseases giving rise to the inaccuracy of quantification results, especially when the age increase, the bias bigger (Cho et al., 2017). Furthermore, the problem of a critical level of degradation in the amount of full-length DNA after conventional bisulfite treatment is yet to be addressed (Meng et al., 2019). In light of various restrictions and challenges mentioned above, looking for other appropriate biomarkers in human blood is of great significance for forensic age estimation.

With the advent of next-generation RNA sequencing (RNA-seq) and bioinformatics approaches, circular RNAs (circRNAs) have emerged as an interesting RNA species (Salzman et al., 2012; Jeck et al., 2013). CircRNA is a class of newly discovered noncoding RNA (ncRNA), presenting as a special covalent loop without a 5′cap or 3′ tail (Li et al., 2018b). Their unique feature enhances their ability to resist the degradation of exonucleases and thus contributes to their stability compared to their mRNA counterparts (Enuka et al., 2016; Zhang et al., 2016). Recent studies have reported that circRNAs have a number of binding sites for the microRNA (miRNA) family acting as the miRNA sponge and interact with RNA-binding proteins (RBPs). They play a role in aging and age-related diseases like Alzheimer’s disease, through interaction with miRNAs and RBPs (Cai et al., 2019). Apart from the properties of resistance to RNase R digestion, miRNA, and RBP sponges, circRNA also exhibits other biological characteristics such as widespread expression, cell-specificity, tissue-specificity, and developmental stage-specific expression patterns (Abu and Jamal, 2016). Its feature of developmental stage-specific expression has been confirmed across various model organisms such as mice (Gruner et al., 2016), flies (Westholm et al., 2014), and elegans (Cheng et al., 2016). Accumulating evidence indicates the involvement of circRNAs in aging, indicating a potential role as biomarkers of the chronological age. Hall et al. (Hall et al., 2017) identified 38 circRNAs that were differentially expressed between day 10 and 40 from RNA-seq profiles of *Drosophila* photoreceptor neurons. After detecting the global profiles of circRNAs in *C. elegans* from the fourth larval stage (L4) through 10-day-old adults, Cortes-Lopez et al. (Cortes-Lopez et al., 2018) found a massive accumulation of circRNA expression levels during aging. Yu et al. and others identified senescence-associated circRNAs by the whole-transcriptome sequencing and discovered circCCNB1 sponges miRNA miR-449a to inhibit cellular senescence by targeting CCNE2 (Yu et al., 2019). Although the fact that circRNAs expressions are observed to accumulate during aging in various organisms and human senescent cells (Haque et al., 2020), research focusing on circRNAs for age prediction has not been carried out yet in the field of forensic. In our preliminary work, we screened a total of 23 age-related circRNAs using univariate linear regression analysis (Wang et al., 2019). In the current study, we introduced other machine learning algorithms, support vector machine (SVM), and lasso regression to conduct in-depth data mining on circRNA sequencing data and screen out more age-related circRNA candidates. Selected age-related circRNAs were validated in a larger sample size and investigated for modeling for age prediction.

In this study, we analyzed circRNA-seq profiles from 13 blood samples of unrelated Chinese aged between 20 and 62 years, focusing on the potential links between the chronological age and the expression of circular RNAs in human blood. The present study aims to build a novel age prediction model based on a subset of age-related circRNAs for forensic application. Machine learning has gained a place in medicine and captured the interest of medical researchers (Cabitza and Banfi, 2018). We used several machine-learning methods to select age-related circRNAs and build age prediction models. Five different models including multivariate linear regression (MLR), regression tree, bagging regression, random forest regression (RFR), and support vector regression (SVR) were established based on five age-associated circRNAs using 100 samples. To the best of our knowledge, this is the first study that uses circRNAs in the blood as indicators together with machine learning methods to develop prediction models for forensic individual age estimation.

## Materials and Methods

### Sample Collection and RNA Isolation

Whole blood samples were collected from 13 healthy volunteers (aged between 20 and 62 years, including 6 females and 7 males) for circRNA sequencing and 30 subjects (aged between 19 and 72 years, including 16 females and 14 males) for RT-qPCR experiment validation (Supplementary Table S2). For age prediction model construction, 100 blood samples from 19–73-year olds including 52 females and 48 males were collected. Written informed consent was obtained from all the volunteers, and our study was approved by the Medical Ethics Committee of Hebei Medical University (No. 20190013).

Peripheral whole blood 10 ml were drawn from subjects by venipuncture and collected in an EDTA-containing vacutainer. Total RNA was isolated immediately using TRIzol reagent (Thermo Scientific, United States) according to the manufacturer’s instructions after blood collection. RNA quantification was conducted on the NanoDrop 1000 (NanoDrop Technologies). RNA integrity was assessed using the RNA Nano 6000 Assay Kit of the Bioanalyzer 2100 system (Agilent Technologies, CA, United States). Isolated RNAs were preserved on the condition of −80°C until reverse transcription.

### High-Throughput Sequencing (circRNA-Seq)

A total amount of 5 μg RNA per sample was used as the input material for RNA sample preparation. First, ribosomal RNA was removed by using the Epicentre Ribozero^TM^ rRNA Removal Kit (Epicentre, United States), and the rRNA free residue was cleaned up by ethanol precipitation. Subsequently, the linear RNA was digested with 3U of RNase R (Epicentre, United States) per μg of RNA. The sequencing libraries were generated by NEBNext^®^ Ultra^TM^ Directional RNA Library recommendations. Barcoded libraries were sequenced at Novogene Co., LTD. (Beijing, China) using the Illumina Hiseq 4000 platform to obtain paired-end 150 nt reads. CircRNA candidates were detected and identified using find_circ (Memczak et al., 2013) and CIRI2 algorithms (Gao et al., 2015). The normalization of contig counts was performed by calculating transcripts per million (TPMs). The normalized expression level = (read counts *1,000,000)/libsize (libsize is the sum of circRNA read counts).

### Reverse Transcription and Quantitative PCR (RT-qPCR)

RNA was reverse transcribed into cDNA using PrimeScript Reverse Transcriptase^TM^ (Takara Bio Inc., Otsu, Shiga, Japan) according to the manufacturer’s protocol. cDNA was obtained using the RT Primer Mix including the oligo dT primer and random 6 mers and was stored at −80°C waiting for further RT-qPCR tests. qPCR reactions were performed using the QuantiNova^TM^ SYBR^®^ Green PCR Kit (Qiagen, Germany) on a 7500 System (Applied Biosystems). Circular RNAs are circular in shape and covalently closed. The primer design is of vital importance for PCR quantitation. The use of divergent rather than convergent primers can selectively detect and quantitate these special RNA molecules (Li et al., 2015). Sequences of primers are available in Supplementary Table S3. 18S rRNA was chosen as reference genes which were stably expressed in blood samples in the pre-test. The delta Ct value (Ct _target_ – Ct _reference_) (ΔCt) represented the circRNA expression. These tests were performed using technical duplicates. PCR products were also electrophoresed on agarose gel and recovered to verify the specificity of primers using Sanger sequencing.

### Selection of Potential Age-Associated circRNAs

An enormous amount of data produced by the next generation sequencing makes the pre-selection process very important and challenging. In an attempt to limit the quantity of predictors which may save both time and cost, three statistical methods were conducted to select age-associated markers. According to circRNA-seq data, an adjusted *p*-value lower than 0.01 (the false discovery rate method) after the Spearman’s correlation analysis was regarded as a criterion to identify the age-associated circRNAs using IBM SPSS statistics software for Windows, version 21.0. Lasso regression was implemented for feature selection with an alpha value set in a range from 0.0005 to 1 within Python. Another feature selection approach SVM was performed in Python using the ‘*LinearSVC*’ command with the ‘*C*’ value set to 0.0202. The Spearman correlation coefficients (S.rho) were then calculated again for 30 validation samples according to qPCR results to validate age-dependent circRNAs. CircRNA host gene analyses were performed on *string-db.org* and the gene database of www.ncbi.nlm.nih.gov *.*

### Age Prediction

The R project for statistical computing software version 4.0.0 was employed to conduct five algorithms: Multivariate Linear Regression (*lm*), regression tree (*rpart*), bagging regression (*ipred*), random forest regression (*randomForest*), and support vector regression (*rminer*). The RMSE and MAE values between chronological and predicted ages were used as performance metrics for the final age prediction models using the five above-mentioned algorithms. Two important parameters *mtry* referring to the number of features to be considered at each split and *ntree* standing for the number of trees in a forest were set to 5 and 300, respectively. VIM is one of the methods in capturing the patterns of dependency between variables and response in the form of a single number. It can essentially be used in many prediction methods but is particularly effective for black-box methods which (in contrast to, say, generalized linear models) yield less interpretable results (Shen et al., 2019). We ranked VIM according to the accuracy, MSE, and standard errors, separately.

## Results

### Selection of circRNAs Differentially Expressed During Aging

**FIGURE 1:**
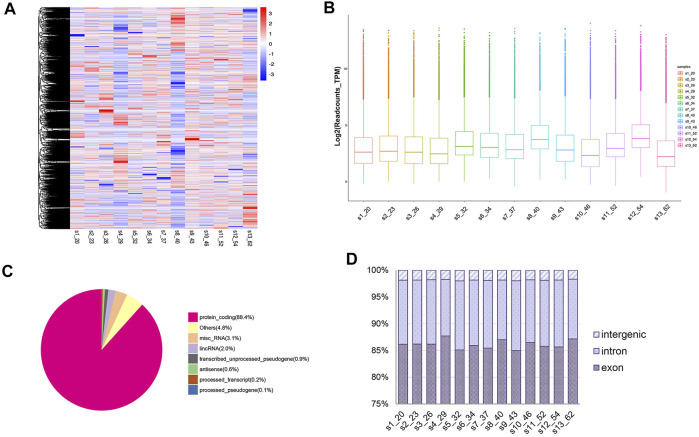
Landscape of circRNA profiles in sequencing samples. **(A)** Heatmap of circRNA expressions across all 13 samples; **(B)** Box plots of circRNA TPM readcounts between different ages. **(C)** Classification of mapped reads taking sample 1 (20 years old) for example. **(D)** Genomic features of circRNAs in each sample.

A total of 45,697 circRNAs were identified by circRNA high-throughput sequencing [56,330 circRNAs were identified in our published data that circRNA identification was performed using the find_circ algorithm alone (Wang et al., 2019)] (Figure 1A). Our results demonstrated a high abundance of circRNA in human peripheral whole blood with an average of 26,719 read counts for each individual. To further illustrate distribution of circRNA levels between different ages, we plotted circRNA log_2_ TPM readcounts for all samples (Figure 1B). Additionally, circRNAs were distributed across various genomic regions but most commonly from protein coding regions, where over 80% of circRNAs originated (Figure 1C). We also observed exons and introns accounting for a higher proportion (more than 95% in each sample) than intergenic regions (Figure 1D).

To identify age-correlated circRNA candidates, we introduced three methods of feature dimension reduction, including false discovery rate (FDR), lasso regression (LASSO), and support vector machine (SVM). The Spearman’s correlation coefficient (S.rho) was calculated to identify the correlation between the age and circRNA expression level (TPM value) for each single circRNA. For FDR-adjusted method after Spearman correlation analysis, 14 circRNAs were considered as age-related markers. Eight circRNAs were selected using the lasso regression method. The SVM method selected 10 circRNAs from more than 40,000 circRNAs (see Methods & Materials). A subset of these 28 circRNAs was chosen for age-associated circRNA candidates for further RT-qPCR validation in a cohort of 30 samples (Supplementary Table S1). There were four overlapped circRNAs screened both by LASSO and SVM methods, including hsa_circ_0002454, hsa_circ_0006117, hsa_circ_0014606, and hsa_circ_0032800.

Information of 30 validation samples is listed in Supplementary Table S2. Spearman correlation analyses showed five circRNAs selected by RNA-seq were in good agreement with RT-qPCR quantifications. All five age-related circRNAs showing a statistically different change by qPCR were upregulated during aging revealing an overwhelming bias for the upregulation of circRNAs during aging. Note that the lower the ΔCt-value, the higher is the circRNA expression. Among them, circRNA hsa_circ_0086306 showed higher Spearman correlation coefficients (S.rho = −0.456), followed by hsa_circ_0015789 (S.rho = −0.453). Derived genes of the 5 age-related circRNAs encode several proteins or involve in multiple pathways. The feature description of 5 age-associated circRNAs is listed in Table 1. Finally, 5 age-correlated circRNAs were selected for further age estimation modeling using an independent cohort of 100 samples.

**TABLE 1:** Final circRNA markers for the age prediction models. Description of their features, originated gene name, and tendency during aging.

circRNA ID	Gene	Full gene name	Chr.	Length (nt)	Trend
hsa_circ_0015789	DENND1B	DENN domain containing 1B	1	477	Up
hsa_circ_0086306	UHRF2	Ubiquitin like with PHD and ring finger domains 2	9	297	up
hsa_circ_0002454	DNAJC6	DnaJ heat shock protein family (Hsp40) member C6	1	350	up
hsa_circ_0000524	RBM23	RNA-binding motif protein 23	14	189	up
hsa_circ_0004689	SWT1	SWT1 RNA endoribonuclease homolog	1	469	up

### Development of Age Prediction Models Using Different Algorithms

**FIGURE 2:**
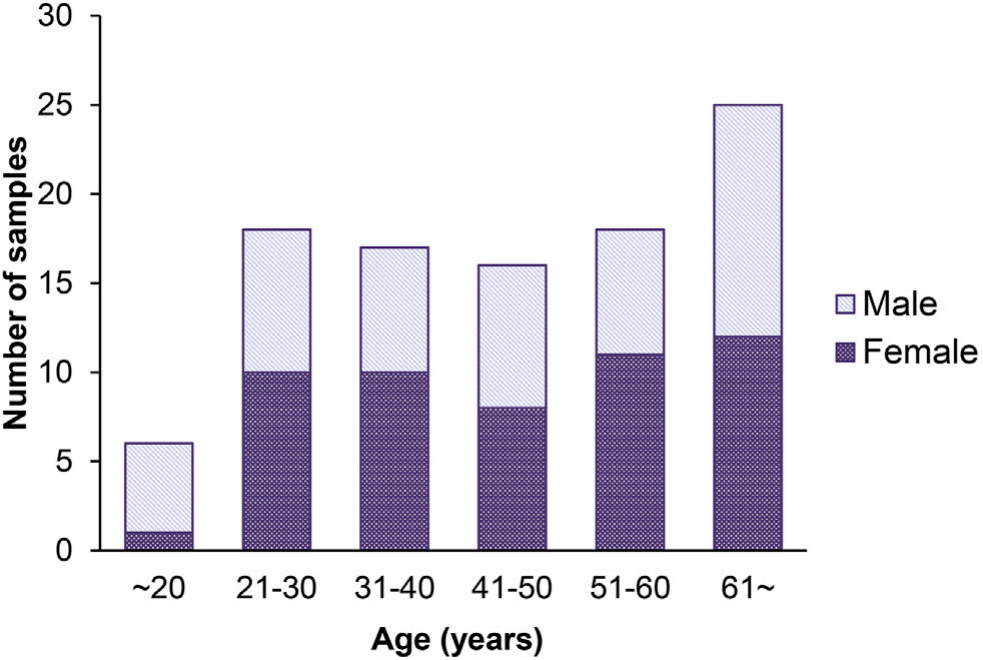
Histogram of the age distribution for 100 healthy volunteers. The *x*-axis represents the chronological age of the individuals (age unit is years), and the *y*-axis (counts) represents the number of individuals.

**FIGURE 3:**
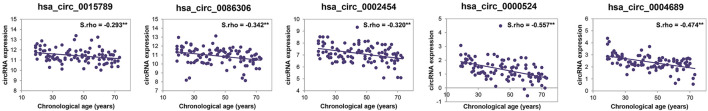
Scatter plots of the ΔCt-value versus age for 5 age-related circRNAs in 100 samples for modeling.

**FIGURE 4:**
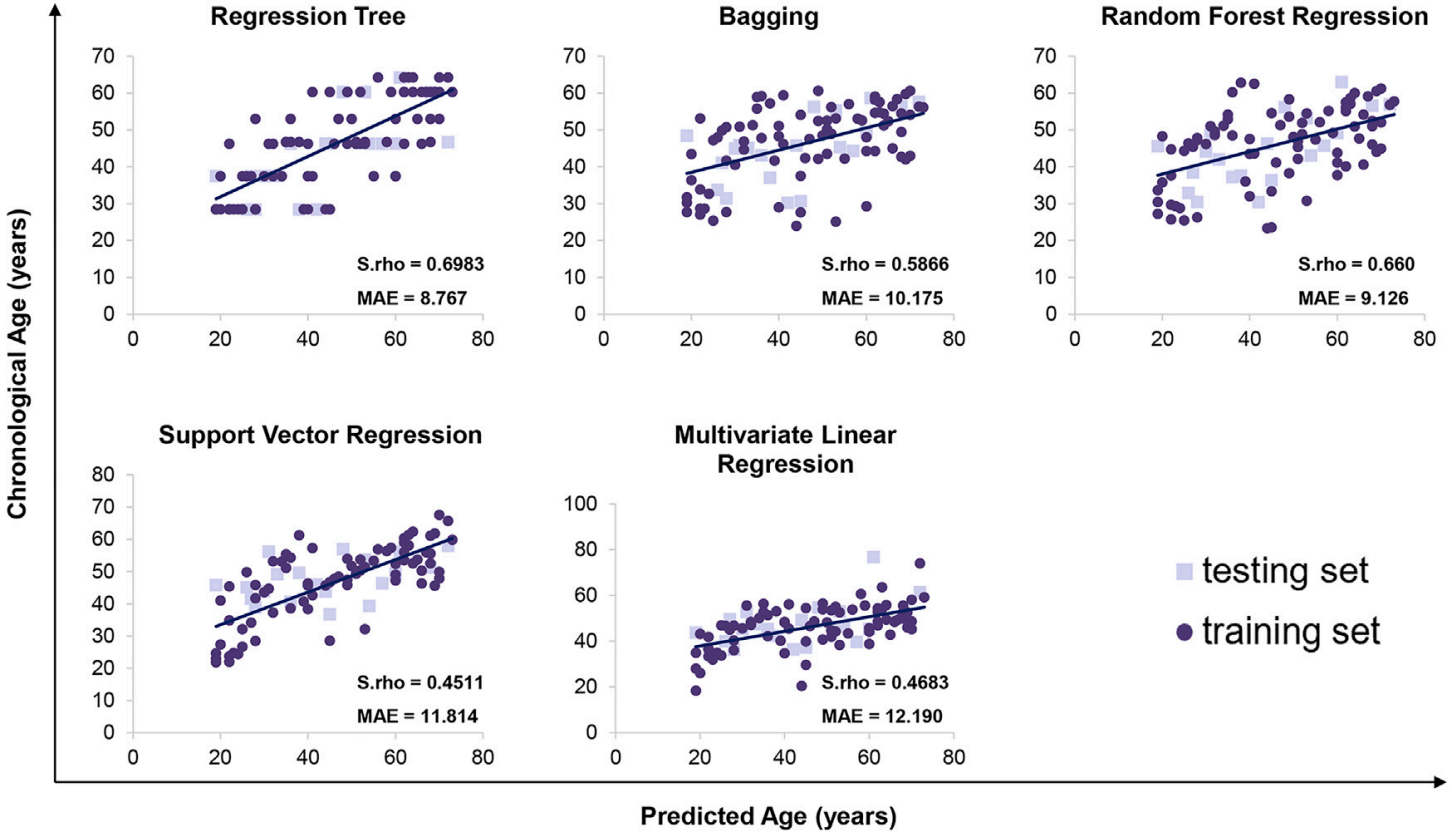
Predicted versus chronological ages using regression tree, bagging, random forest regression, support vector regression, and multivariate linear regression.

We detected 5 age-related circRNAs in 100 blood samples aged between 19 and73 years old using the RT-qPCR strategy (Figure 2). The scatter plots of 5 circRNAs in 100 samples are displayed in Figure 3. To provide an unbiased estimate of a predictive accuracy for age, various attempts had been made to develop better models to predict the age. We adopted five different algorithms, including Multivariate Linear Regression (MLR), bagging regression, regression tree, random forest regression (RFR), and support vector regression (SVR). Age prediction models were fit based on 5 age-related circRNAs. The whole blood dataset was randomly split into the training subset (80%, *n* = 80) and the testing subset (20%, *n* = 20). Results uncovered that the regression tree and the RFR models showed the highest and similar accuracy with MAE values of 8.767 years (S.rho = 0.6983) and 9.126 years (S.rho = 0.660) in the testing subset, while the SVR model reached the highest accuracy in the training subset (MAE = 8.367, S.rho = 0.7423) but performed poorly in the testing subset (MAE = 10.175, S.rho = 0.4511). Other models showed medium MAE values in the testing subset: 10.175 years for bagging and 12.190 years for the MLR (Table 2). The relationship between the chronological and the predicted age using different algorithms is displayed in Figure 4. The RFR model fitted 30% (6/20) of individuals within a ±5 year error range, while 50% (10/20) within a ± 10 year error range. Additionally, the variable importance measure (VIM) ranking the variables (i.e., the features) with respect to their relevance for prediction is a byproduct of random forest (Shen et al., 2019). As an illustration, the ranks of variable importance measures according to accuracy, MSE, and standard errors are displayed in Supplementary Figure S1 separately.

**TABLE 2:** Comparison of five prediction models.

Models	Training set (*n* = 80)			Testing set (*n* = 20)		
	MAE (years)	RMSE (years)	S.rho	MAE (years)	RMSE (years)	S.rho
Tree	9.343	11.162	0.7405	8.767	10.584	0.6983
Bagging	12.311	14.650	0.4888	10.175	12.04	0.5866
RFR	12.442	14.543	0.4975	9.126	11.168	0.660
SVR	8.367	11.187	0.7423	11.814	13.716	0.4511
MLR	11.925	13.690	0.5662	12.190	13.825	0.4683

### Age and Sex Analyses

In order to further evaluate the predictive error, samples were divided into five age groups: 20s or less, 30, 40, 50, and 60s or more. In line with previous studies, the MAE increased with age (Naue et al., 2017). In our study, older individuals (more than 60 years old) presented an increased deviation between the chronological and predicted age compared to younger groups (less than 40 years old), and the predicted age of elders was prone to be underestimated. Specifically, the tree model, for example, the MAE was 7.985 years for the youngest age group (20s or less), 7.656 years for 30s, 7.335 years for 40s, 8.604 years for 50s, and 12.493 years for the oldest (60s or more).

The observation that differences between sexes exist was found by the previous study (Fehlmann et al., 2020). Whether sex effects did have an impact on the accuracy of age prediction models in our study, we conducted an analysis on female and male samples for model fitting separately. Similarly, all five models in the current study significantly yielded smaller prediction MAE values for males than females in the training subset, and 3 out of 5 models in the testing subset are as shown in Table 3. For instance, the regression tree model reached an MAE of 6.133 years (S.rho = 0.882) in males but 10.923 years in females (S.rho = 0.562). Results indicated age prediction models for female samples were less accurate than those for male samples.

**TABLE 3:** Age prediction performance of different models between female and male groups.

Models	Training set (*n* = 80)						Testing set (*n* = 20)					
	Female (*n* = 41)			Male (*n* = 39)			Female (*n* = 11)			Male (*n* = 9)		
	MAE (years)	RMSE (years)	S.rho	MAE (years)	RMSE (years)	S.rho	MAE (years)	RMSE (years)	S.rho	MAE (years)	RMSE (years)	S.rho
Tree	10.284	12.226	0.654	**8.352**	**9.975**	**0.809**	10.923	12.634	0.562	**6.133**	**7.338**	**0.882**
Bagging	13.355	15.569	0.368	**11.213**	**13.617**	**0.609**	10.192	12.742	0.526	**10.154**	**11.122**	**0.660**
RFR	13.692	15.559	0.373	**11.129**	**13.392**	**0.624**	9.531	11.919	0.599	**8.630**	**10.175**	**0.732**
SVR	9.168	11.947	0.673	**7.524**	**10.327**	**0.807**	11.380	13.649	0.497	12.345	13.798	0.409
MLR	12.385	13.963	0.502	**11.441**	**13.398**	**0.619**	10.804	13.188	0.51	13.883	14.566	0.461

The bold values means models with a lower MAE value.

## Discussion

The development of molecular methods for age prediction is valuable when the human specimens such as bloodstains are retrieved from crime scenes without morphological age features. Considerable progress of the molecular biology of aging has been made in the last few decades. Using a variety of age-associated biomarkers in blood has emerged as a useful method for age estimation. Nevertheless, estimating age accurately and reliably from molecular biomarkers is still a challenge because identifying minimal numbers of biomarkers that provide maximal age information is extremely tough when analyzing forensic samples as the nature of forensic DNA or RNA being of lower quality and quantity (Zubakov et al., 2016). New insights into discovering novel biomarkers with the property of strong correlation of aging and stability need more attention within the forensic genetic community.

In this study, we provided potential age-associated markers in human blood, namely, circRNA due to its property of highly stable and developmental stage-specific expression patterns. Recently, some analyses conducted by forensic researchers have focused on the capacity of circRNAs to identify body fluids due to their tissue-specificity (Dong et al., 2016). We also noticed that circRNAs have been studied in aging and age-related diseases across different species, revealing a global accumulation of the circRNA expression level during aging. Additionally, the unique feature of circRNAs enhances their stability and thus might be more suitable for forensic-degraded samples seen commonly. To substantiate this assumption, we identified age-dependent circRNA markers from the human peripheral whole blood by means of circRNA next-generation sequencing and RT-qPCR and developed age-predictive models using different machine learning algorithms. To the best of our knowledge, this is the first one to predict an individual’s age using circRNA biomarkers. Our study is non-trivial; we opened a new avenue for forensic age prediction analysis using a novel biomarker in human blood, which would be an important topic for future investigation.

In recent years, sequencing technological advances in monitoring the gene expression have given rise to a dramatic increase in gene expression data. Selecting age-associated circRNAs from a giant gene dataset of RNA-seq profiles (approximately over 40,000 circRNAs were identified) made the current study difficult. Machine learning techniques are known to be excellent at discarding and compacting redundant information from gene expression data. The introduction of machine learning algorithms helped us to select variables from 45,697 down to 28. Five out of 28 age-dependent circRNAs finally selected all showed positive correlations with the chronological age. This expression trend was in accordance with the previous research in other organisms that most circRNA expression levels dramatically increase during aging (Mahmoudi and Cairns, 2019). Though there has been no existing evidence yet indicating that circRNA expression levels of human peripheral whole blood are changing with increasing age, recent reports showed that circRNA expression levels change in the elders’ brains with aging and play a vital role in the development of neurodegenerative diseases such as Alzheimer’s disease (AD) and Parkinson’s disease (PD) (Constantin, 2018; Zhang et al., 2018; Hanan et al., 2020). Furthermore, Shahnaz Haque et al. (Haque et al., 2020) assessed the circRNA expression in aging human blood and found that *circFOXO3* and *circEP300* demonstrated differential expression in one or more human senescent cell types, providing indirect evidence for circRNA as a promising indicator for age estimation.

Selected circRNA candidates in our study were derived from five different genes (*DENND1B, UHRF2, DNAJC6, RBM23,* and *SWT1*) and might involve in several important biological processes. We then undertook a comprehensive analysis. As expected, these five genes code for important proteins or involve in biological metabolic processes. The gene *DENND1B* encodes the guanine nucleotide exchange factor (GEF) for *RAB35* that acts as a regulator of T-cell receptor (TCR) internalization in TH2 cells which functions as a regulator in the process of childhood asthma (Fauna et al., 2017). The gene *UHRF2* encodes a protein that is an intermolecular hub protein in the cell-cycle network (Mori et al., 2002). Through cooperative DNA and histone binding, it may contribute to tighter epigenetic control of the gene expression in differentiated cells. Recent research found that *UHRF2* can promote the DNA damage response (Wang et al., 2018). The gene *DNAJC6* belongs to the evolutionarily conserved *DNAJ/HSP40* family of proteins, which regulate the molecular chaperone activity by stimulating the ATPase activity. This gene plays a role in clathrin-mediated endocytosis in neurons (Yim et al., 2010). The gene *RBM23* encodes probable RNA-binding protein and may be involved in the pre-mRNA splicing process (Dowhan et al., 2005). *SWT1* is a protein coding gene that involves in diseases including kidney sarcoma and Wilms tumor 1. Notably, the function of circRNAs or their host genes can provide some clues for future selection of biomarker candidates. Some circRNAs were confirmed to regulate biogenesis, and some senescence-associated genes might become a clue for the selection of age-dependent indicators, for instance, gene *Foxo3* circular RNA was highly expressed in heart samples of aged patients and mice reported by William W. Du et al. (Du et al., 2017). *Foxo3* was found with different expressions in one or more human senescent cell types in the study of Shahnaz Haque et al. (Haque et al., 2020). In contrast, some of the aforementioned circRNAs play a key role in the pathogenesis of tumors or diseases, such as *DENND1B* and *SWT1*. The effect of the disease state on age predictions should be considered for further investigation because it is important to build a robust predictive model using biomarkers that would not present differential expression due to the disease status. As models described in the study by Athina Vidaki et al. (Vidaki et al., 2017), they analyzed the effect of different diseases on age prediction that schizophrenia presented the lowest age prediction error, while anemia demonstrated the lower relation with age, indicating that it becomes evident that the error is much higher for blood-related diseases when analyzing separately, samples suffering from blood- vs. non–blood-related diseases.

Among the multiple machine learning algorithms adopted for the present study, the regression tree and RFR models showed the highest and similar accuracy in the testing subset. The SVR model reached the highest accuracy in the training subset but had a lower accuracy in the testing subset, which might be due to overfitting and loss of generalizability. These estimation errors were higher than previous predictive models; therefore, using other biomarkers (Cho et al., 2017; Dias et al., 2020) might be due to the small sample size. Small sample sizes might lead to biased screening of age-related biomarkers, which would explain why we screened age-associated circRNAs with only four overlapped using different methods. The limited sample size might restrict our models’ validity to a certain extent. The possibility that these age-related biomarkers might not be able to apply in other groups of different ethnic origins is due to the very small cohorts employed for the initial RNA-sequencing analysis, which limits the value of this study to the Chinese population alone. This also restricts the potential impact of childbearing on women’s data as circRNA profiles in human granulosa cells were reported age-related and potentially reflecting decreased oocyte competence during maternal aging (Cheng et al., 2017). Besides, although machine learning algorithms seem to achieve a relatively high accuracy using a subset of weak predictors, the prediction accuracy of models presented here is needed to be further improved to satisfy forensic practice, which means more robust, stable, and higher age-related circRNA biomarkers need to be found in our future work. Our models performed better in young subjects but poorly for elders over 60 years old. We suppose that the deviation might attribute to the fact that adults and elders suffer from more confounding factors in the aspect of medication, smoking, or alcohol, which are not easily accessible for children and adolescents, as previously reported (Li et al., 2018a). We are aware of the other one weakness in the current study that our sample set did not incorporate children and adolescents (a population sample ranging from 19 to 73 years old was used). Therefore, future studies are needed to explore age-specific biomarkers, namely, under-aged-specific and adult-specific predictive indicators. Focusing on when these biomarkers occur and when they lose functions or their periodical changes during an individual’s life span also need to be investigated precisely in further research. Additionally, aging can be also affected by the sex effect as females tend to live longer than males, and the occurrence of certain diseases are sex-specific, for instance, neurodegenerative diseases such as Parkinson’s disease are more prevalent in males (Cerri et al., 2019). Sex analysis showed all five models in our study significantly yielded smaller prediction MAE values for males than females in the training subset, indicating age prediction models for female samples were less accurate than male samples. This might be due to female hormones such as estrogen, which can protect females from some diseases, for example, bone loss increases dramatically in women after menopause (Streicher et al., 2017). The presence of a single X chromosome in males (rather than two in females) might also explain why males are more susceptible to genetic diseases linked to the X chromosome such as hemophilia (Shoukat et al., 2020).

It is worth asking what underlying mechanisms might give rise to the increased levels of circRNAs with aging. Some people hold that the age-related increasing trends for circRNAs are reflective of age-accumulation more so than specific regulation. Such an assumption is based on studies of multiple animals discovering that the increased abundance of circRNAs during aging was found to be largely independent of the gene expression from their host due to their lack of 3′ or 5′ end (Knupp and Miura, 2018). In addition to the contribution of circRNA stability, some researchers suppose that increased circRNA biogenesis due to age-related changes in alternative splicing might play a role (Kramer et al., 2015). These results prompt us that it might be necessary to figure out the functions of circRNAs in aging and mechanisms contributing to circRNA changes during aging.

In the present study, we first constructed age prediction models using novel biomarkers, circular RNAs. Our analysis of blood-derived circRNAs provides insights into changes in circRNA abundance dependent on age and is an exploration for circRNAs as potential biomarkers to be applied in forensic practice in trap future. It is convinced that the accuracy of models can be improved in our future work through newly age-related circRNAs discovered, algorithms optimized, sample sizes enlarged, and a wide age range included. In a word, based on our results, we argue that the prediction of the chronological age utilizing age-dependent changes of specific circRNAs is a promising application and will become an increasing field of interest.

## Data Availability

The datasets presented in this study can be found in online repositories. The names of the repository/repositories and accession number(s) can be found in the article/Supplementary Material.
