# Supplementary material for: Circular RNA as a Potential Biomarker for Forensic Age Prediction
Source: Front Genet. 2022 Feb 7;13:825443. doi: 10.3389/fgene.2022.825443 (PMC8858837; doi:10.3389/fgene.2022.825443)
Supplement: Supplementary file 1 [file DataSheet4.zip › SUPPLEMENTARY_MATERIAL/supplementary materials.DOCX]

Supplementary Material

# Supplementary Figures and Tables

## Supplementary Figures

**
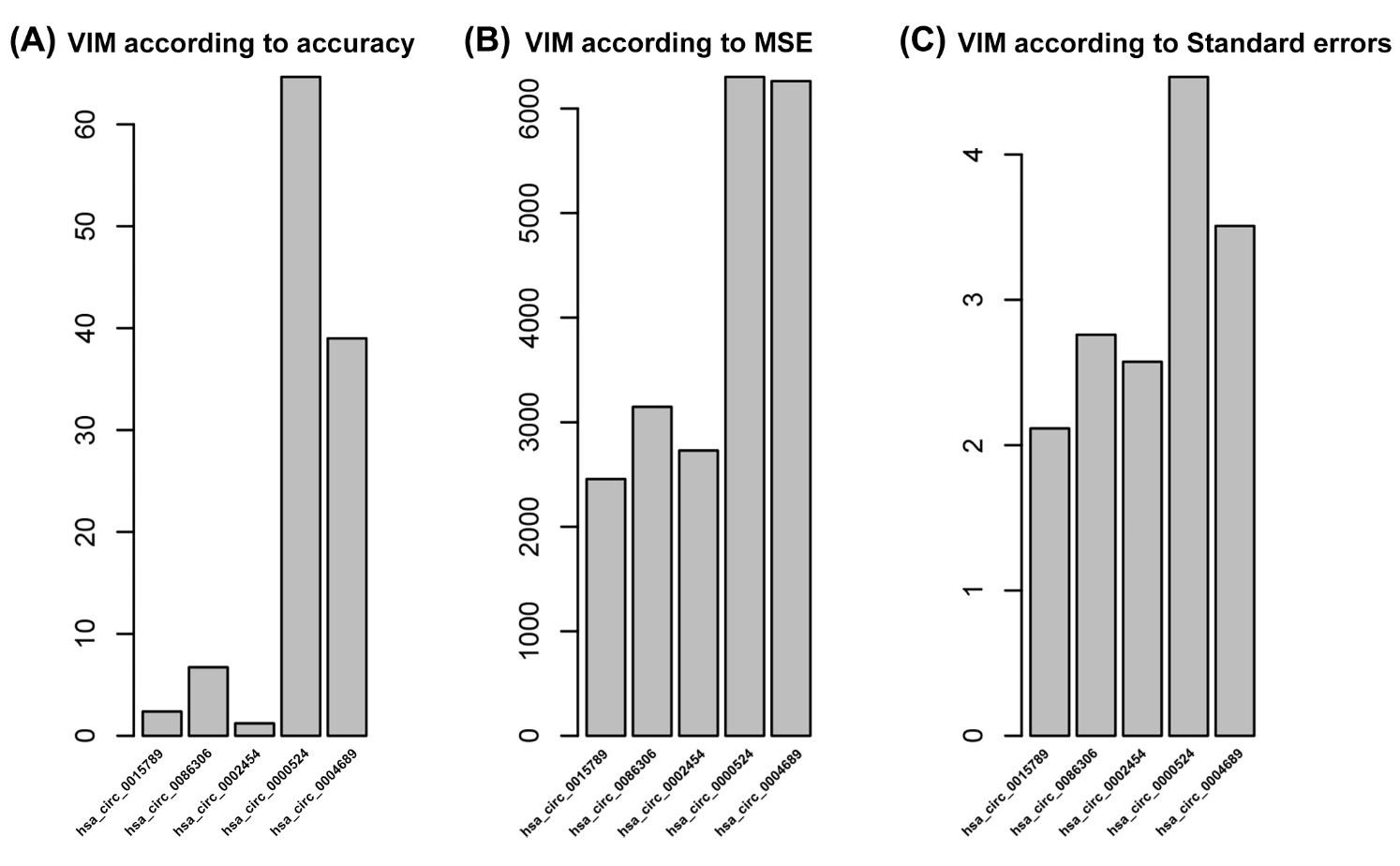
**

**Supplementary Figure 1.** Variable importance measures (VIM) of RFR model according to **(A)** accuracy, **(B)** MSE and **(C)** Standard errors.

## Supplementary Tables

**Supplementary Table 1. 28 circRNAs selected by three separate approaches**

| **FDR-adjusted** | **Lasso regression** | **SVM** |
| --- | --- | --- |
| hsa_circ_0015789  hsa_circ_0079940  hsa_circ_0006517  novel_circ_0018323  hsa_circ_0060143  novel _circ_0038821  hsa_circ_0086306  novel_circ_0046335 hsa_circ_0005407  novel_circ_0090929 hsa_circ_0001985  novel_circ_0042020  novel_circ_0072384  novel_circ_0089458 | hsa_circ_0000745  hsa_circ_0001360  hsa_circ_0002454*  hsa_circ_0006117*  hsa_circ_0009154  hsa_circ_0014606*  hsa_circ_0021350  novel_circ_0032800* | hsa_circ_0000095  hsa_circ_0000524  hsa_circ_0001400  hsa_circ_0002124  hsa_circ_0002454*  hsa_circ_0004689  hsa_circ_0006117*  hsa_circ_0008285  hsa_circ_0014606*  novel_circ_0032800* |
|  |  |  |

*: Identical circRNAs selected by different methods

**Supplementary Table 2. Information of circRNA-seq samples and validation samples**

| **Sample_ID** | **Age (years)** | **Gender** | **Experiment** |
| --- | --- | --- | --- |
| S1_20 | 20 | Male | Sequencing |
| S2_23 | 23 | Male | Sequencing |
| S3_26 | 26 | Female | Sequencing |
| S4_29 | 29 | Male | Sequencing |
| S5_32 | 32 | Female | Sequencing |
| S6_34 | 34 | Female | Sequencing |
| S7_37 | 37 | Male | Sequencing |
| S8_40 | 40 | Male | Sequencing |
| S9_43 | 43 | Female | Sequencing |
| S10_46 | 46 | Male | Sequencing |
| S11_52 | 52 | Male | Sequencing |
| S12_54 | 54 | Female | Sequencing |
| S13_62 | 62 | Female | Sequencing |
| val_s1_19 | 19 | Male | RT-qPCR |
| val_s2_19 | 19 | Female | RT-qPCR |
| val_s3_20 | 20 | Female | RT-qPCR |
| val_s4_20 | 20 | Male | RT-qPCR |
| val_s5_22 | 22 | Female | RT-qPCR |
| val_s6_31 | 31 | Male | RT-qPCR |
| val_s7_29 | 29 | Female | RT-qPCR |
| val_s8_30 | 30 | Female | RT-qPCR |
| val_s9_29 | 29 | Female | RT-qPCR |
| val_s10_30 | 30 | Male | RT-qPCR |
| val_s11_40 | 40 | Female | RT-qPCR |
| val_s12_41 | 41 | Male | RT-qPCR |
| val_s13_40 | 40 | Female | RT-qPCR |
| val_s14_40 | 40 | Male | RT-qPCR |
| val_s15_39 | 39 | Male | RT-qPCR |
| val_s16_51 | 51 | Female | RT-qPCR |
| val_s17_49 | 49 | Female | RT-qPCR |
| val_s18_49 | 49 | Male | RT-qPCR |
| val_s19_48 | 48 | Female | RT-qPCR |
| val_s20_51 | 51 | Male | RT-qPCR |
| val_s21_61 | 61 | Male | RT-qPCR |
| val_s22_56 | 56 | Female | RT-qPCR |
| val_s23_63 | 63 | Male | RT-qPCR |
| val_s24_60 | 60 | Female | RT-qPCR |
| val_s25_60 | 60 | Male | RT-qPCR |
| val_s26_68 | 68 | Female | RT-qPCR |
| val_s27_69 | 69 | Female | RT-qPCR |
| val_s28_70 | 70 | Male | RT-qPCR |
| val_s29_72 | 72 | Female | RT-qPCR |
| val_s30_68 | 68 | Male | RT-qPCR |

**Supplementary Table 3. Primer sequences of 28 age-related circRNAs for RT-qPCR verification**

| **CircRNA ID** | **Forward Primer (5’ to 3’)** | **Reverse Primer (5’ to 3’)** | **Length** |
| --- | --- | --- | --- |
| **18S rRNA** | ATCCTCAGTGAGTTCTCCCG | CTTTGCCATCACTGCCATTA | 106 bp |
| **hsa_circ_0015789** | ACTGCTGTTACCTTCCCTGG | TTCAGAACTTGCTCACAGGC | 196 bp |
| **hsa_circ_0086306** | AGGAATACTGGGGTTCTGAAGG | ACACAGGTCACATTCAGGGT | 149 bp |
| **novel_circ_0018323** | AAAGGAGCAGCATGGGTTAC | GCAGACAATCTTGACTGAAGC | 192 bp |
| **hsa_circ_0006517** | CTGGACGCTGAGATTGAC | ATGGAAGGGCACTGGAAT | 135 bp |
| **hsa_circ_0002454** | TGTGATGTCCTTTCCTCTGGA | CTGGAGATGAGGCACCCG | 166 bp |
| **hsa_circ_0000524** | GGATTATCCTAGCAATACCACCAG | TCAGATGCCATCCTGTCAGATT | 105 bp |
| **hsa_circ_0004689** | GCTGTGAGGGATATTCTTGAAGG | CTTGAGCAAAGGTCTGTGGAAG | 152 bp |
| **hsa_circ_0079940** | TTACATGCTTGCCTTGGAT | CATATTCGGTCTGGTGGAG | 111 bp |
| **hsa_circ_0060143** | GCTTGCTGGTCTGATTGT | CTCGTCTGTTAGGTGGATG | 198 bp |
| **hsa_circ_0001985** | TCAGCCGTCCTATGATACCAC | GCAATGCCAAGACATCACTCT | 51 bp |
| **novel_circ_0038821** | TATCCACAATCACACCTCAG | GTTAGGTTGAGTTATTCCTCCT | 150 bp |
| **novel_circ_0046335** | TTGACAAGGATGGATCTCAG | CCAGAAGGCATGTTGACT | 155 bp |
| **novel_circ_0090929** | AGGAGGCTGAATGTAGTAAC | CCAGTTCATATCCACAGTTG | 62 bp |
| **novel_circ_0072384** | TTGATCTGCTGTGCTGAG | CCTACAAGTTCGGCTACC | 181 bp |
| **novel_circ_0042020** | GCCTTCCTTACTGACAGAGATG | GTTCCAACCGCTGTATCACA | 137 bp |
| **hsa_circ_0005407** | AGACTGTTCTGTGCCCTCTT | CCCGGGGAAAGAGTACTGAC | 169 bp |
| **novel_circ_0089458** | TGGGCAGATGGAACGAACAT | ACCATTCTTTGCCTCCAGTTC | 185 bp |
| **hsa_circ_0000745** | AGCTGCGAAGTTCAAGAAATGT | GGAATGCCTGTCCGTTTAGT | 169 bp |
| **hsa_circ_0001360** | TGCAGCTACTCAGATCAACCT | GTTGGGTAATACTGCCGCTG | 98 bp |
| **hsa_circ_0009154** | CCTGAAAGATCAATGACGAGCA | CTCTAGTTGGTATCTTCCTCCTTC | 129 bp |
| **hsa_circ_0014606** | GCAGCAGTTCCAAGATGAGATG | CTGCACTGCCCCATAACAAG | 196 bp |
| **novel_circ_0032800** | AGGACCAGTTCAAGATGCCC | CCTCACTCTCATGAAGGGGT | 155 bp |
| **hsa_circ_0000095** | CTACCTTGTACTGGTTGAAGA | AAGAATGGCATGTGGATACT | 202 bp |
| **hsa_circ_0001400** | GCACAGAGTAGCAGCGAATG | CGTATGCAGATGATGGCCAC | 175 bp |
| **hsa_circ_0002124** | ACCCCATCTCCAGACAAATTATT | TGAGGGGACGAGACAAACTT | 117 bp |
| **hsa_circ_0008285** | GTCGGAGCTTTATTGGGCC | TCCTTTCAACCTTTCCCGTTAAC | 143 bp |
| **hsa_circ_0006117** | ACTTTCCCTCCTTCAGATAAGCA | AGCATTGTTGGCACTGACAC | 88 bp |
| **hsa_circ_0021350** | GCAACAGATCCAGGTACACC | AGCTGCTCCCGTAAACTGAT | 92 bp |
